# Supplementary material for: Surface Microbiome Profiling of Dental Elevators Using Third‐Generation Sequencing: Implications for Infection Control in Dental Practice
Source: Microbiologyopen. 2025 Dec 8;14(6):e70178. doi: 10.1002/mbo3.70178 (PMC12685768; doi:10.1002/mbo3.70178)
Supplement: Supplementary file 1 — customer_backup. [file MBO3-14-e70178-s001.zip › customer_backup/customer_backup/taxa_summary/Taxa_dis/treat/sample/treat.sample.genus.bar.pdf]

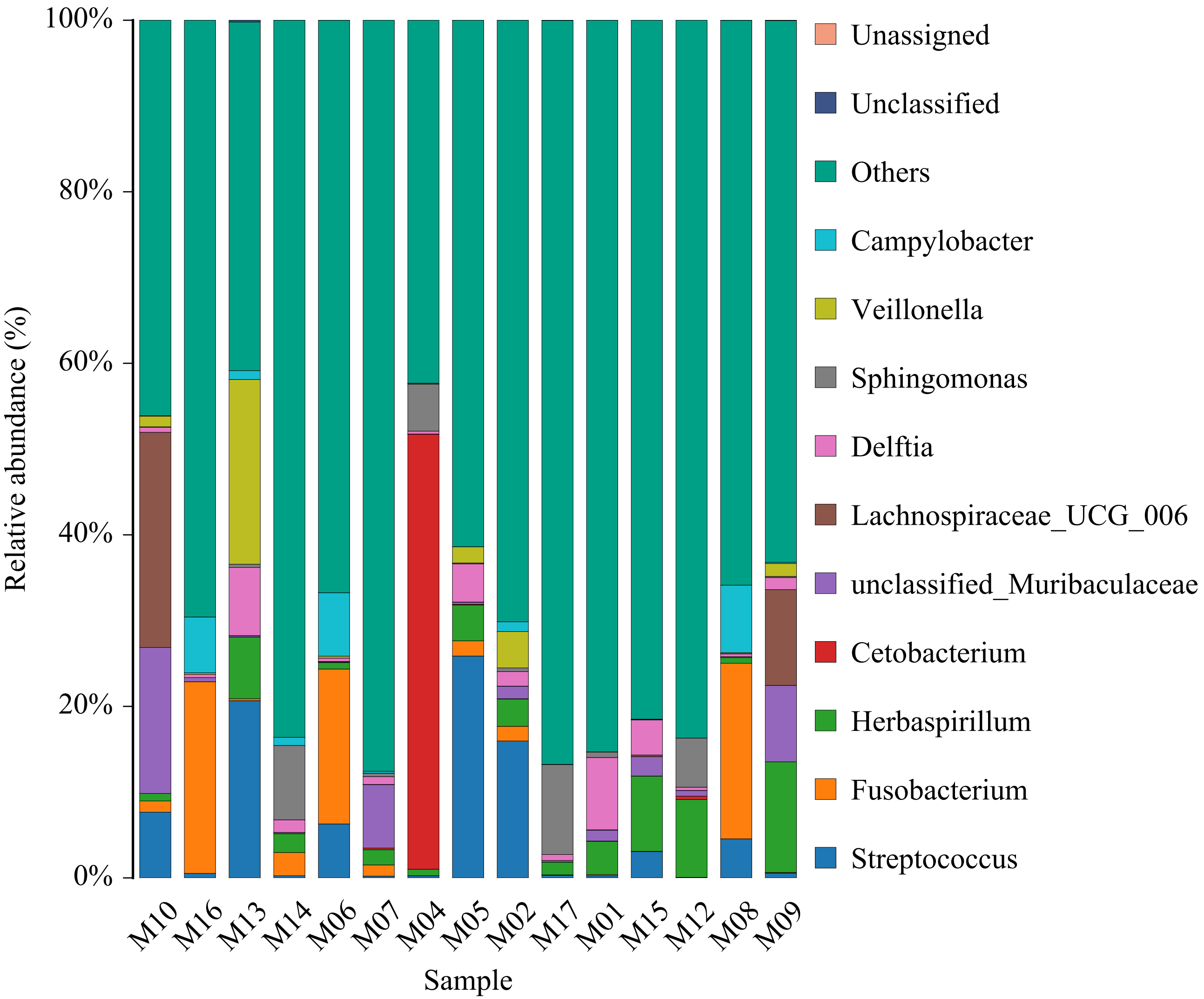

100%
80%
60%
40%
20%
0%
Relative abundance (%)
M10
M16
M13
M14
M06
M07
M04
M05
M02
M17
M01
M15
M12
M08
M09
Sample
Unassigned
Unclassified
Others
Campylobacter
Veillonella
Sphingomonas
Delftia
Lachnospiraceae_UCG_006
unclassified_Muribaculaceae
Cetobacterium
Herbaspirillum
Fusobacterium
Streptococcus
